# Supplementary material for: HIV-Differentiated Metabolite N-Acetyl-L-Alanine Dysregulates Human Natural Killer Cell Responses to Mycobacterium tuberculosis Infection
Source: Int J Mol Sci. 2023 Apr 14;24(8):7267. doi: 10.3390/ijms24087267 (PMC10138430; doi:10.3390/ijms24087267)
Supplement: Supplementary file 1 [file ijms-24-07267-s001.zip › ijms-2308894-supplementary.pdf]

Supplementary Figures

Supplementary Figure S1:

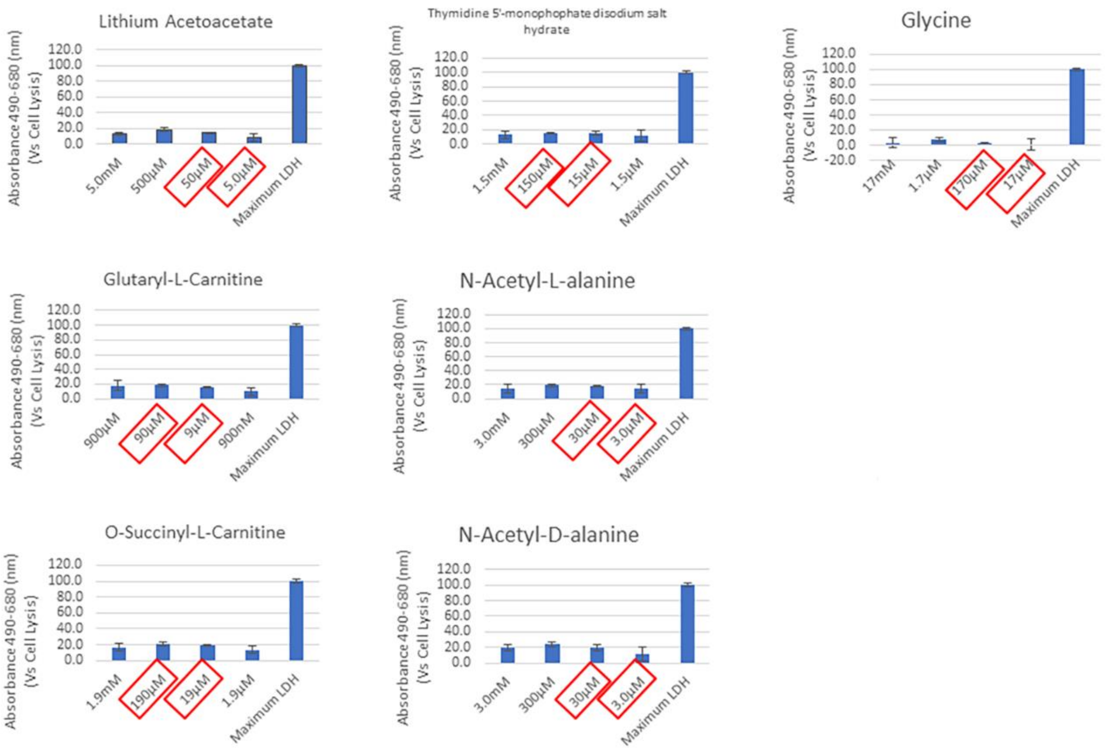

Figure S1. LDH assay was performed to determine the cytotoxicity of metabolic chemicals as described in the Methods section. The cytotoxicity was calculated as the percentage of positive control (the LDH assay kit provided) of the absorbance. For each chemical, two concentrations were chosen (shown in red boxes) for the following experiment shown in Figure 1.

Supplementary Figure S2:

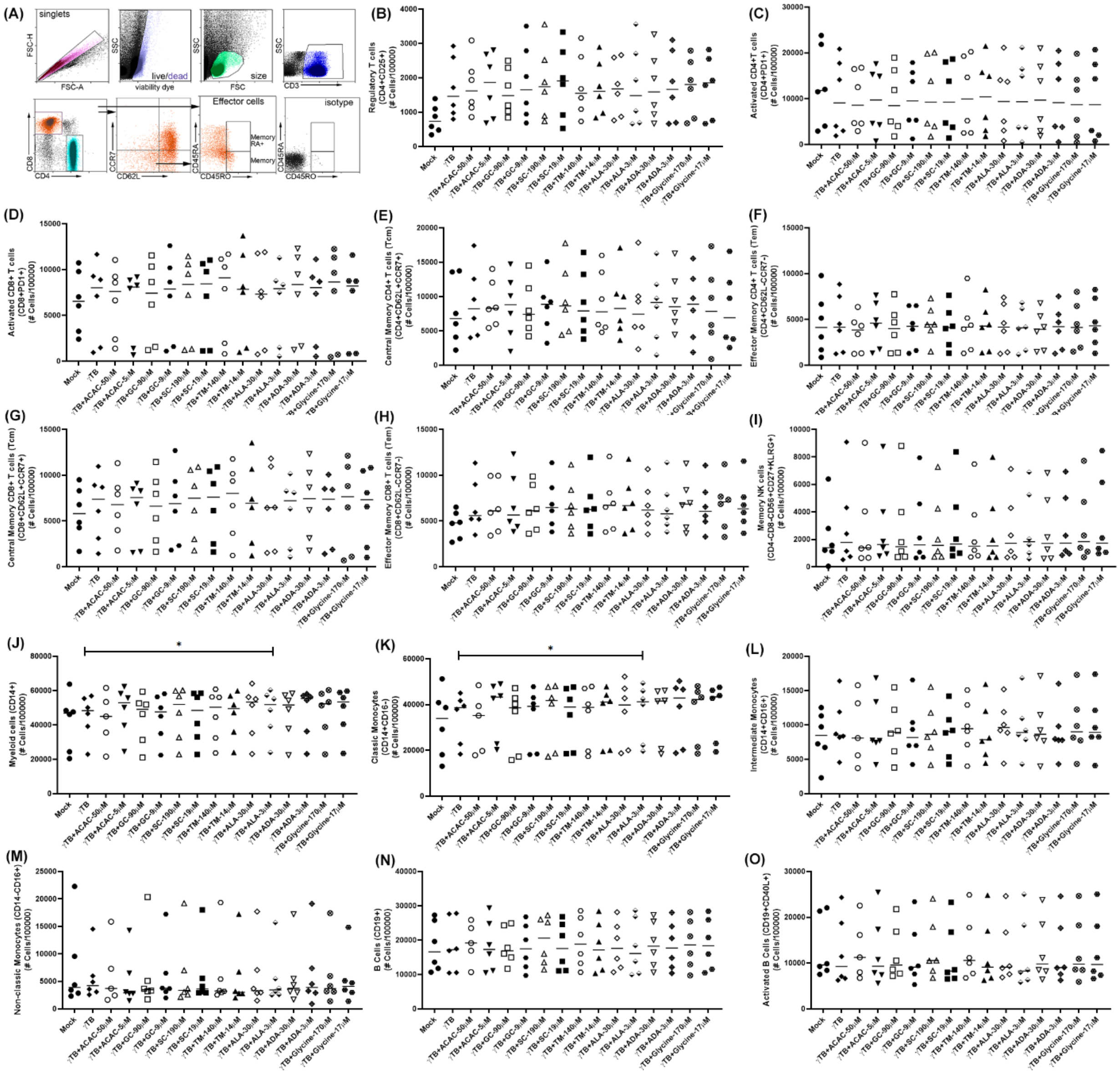

Figure S2. The effects of various metabolites on the expansion of various immune cells and their subpopulations. **(A)** Four panels were designed to stain the immune cells, and one typical panel is shown to describe the gating strategy of various phenotypes of T cells. **(B to O)** Statistical analyses of various immune cells and their subpopulations under different treatments. The paired T-tests were used to determine the differences between the treatment groups. The significant P values are shown (p<0.05).

Supplementary Figure S3:

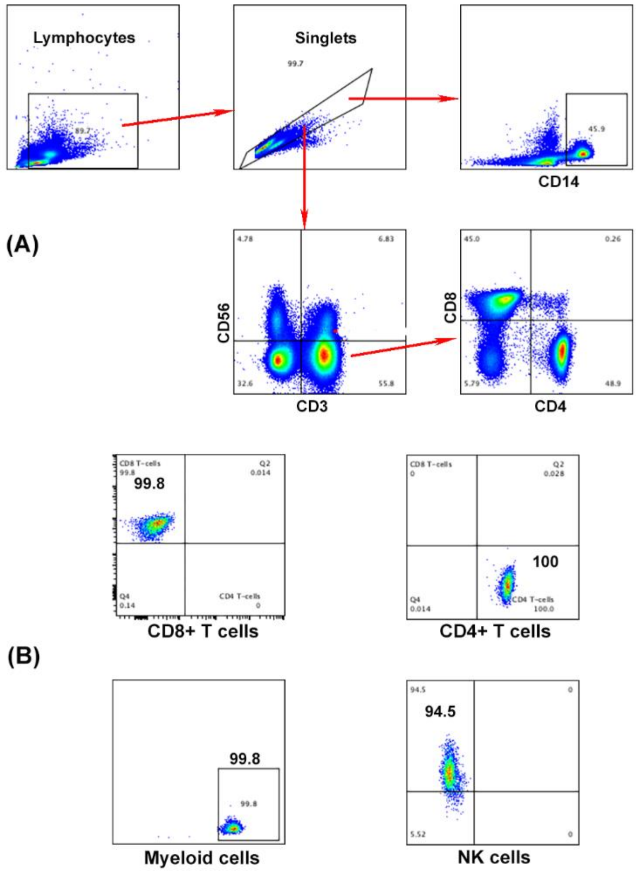

Figure S3. PBMCs from the LTBI+ donors were subjected to cell sorting to collect the CD4+ and CD8+ T cells, myeloid cells, and NK cells. **(A)** The gating strategy for cell sorting. **(B)** The purity of the collected CD4+ and CD8+ T cells, myeloid cells, and NK cells.

Supplementary Table S1: HIV patient information

Patient ID	Age	Gender	Viral load	CD4/CD8 count	date of diagnosis	ART therapy/treatment	naïve: Y or N
4	53	F	20,800	244/1548	2011	Epzicom/ Isentress	N
22	30	M	177,460	518/697	2012	Stribild	N
32	23	M	340	761/999	2012	N/A	Y
38	26	M	1530	871/1154	2012	N/A	Y
40	25	M	212,810	70/530	2012	Truvada/Kaletra/Mepron	N
69	39	M	124,720	90/644	2001	N/A	N
80	32	M	31,430	292/997	2013	Tivicay/Truvada	
83	45	M	90,040	68/1414	2013	N/A	Y

Supplementary Table S2: Primer sequences used in Figure 5.

Name	Sequence
IL21R-F	CTCGTCTGCTACACCGATTAC
IL21R-R	CTCGTCCTTCAGCTCTTCATAC
NKp46-F	TACTGCTCAAGGAGGGAAGA
NKp46-R	AGACCAGGCATGGTTGTTATAG
NKG2D-F	CAGCAAAGAGGACCAGGATTTA
NKG2D-R	GTTAGTAGGTTGGGTGAGAGAATG
KLRC1-F	GATTTACCATCAGCTCCAGAGAA
KLRC1-R	GTTACCACAGAGGCCATTAAGA
KLRG1-F	AGAGACTCACACCTCCTTGT
KLRG1-R	CCTCAGACCAATCCAGCAAA
KLRD1-F	GTACCGGTGCAACTGTTACT
KLRD1-R	CTGAAGCAGGCTGGATTTCT
DNAM1-F	AGGAGAAGGAGAGAGAGAAGAG
DNAM1-R	GATTGGTAGGTTGACTGGTAGAG
2B4-F	CAGCATACAGACGAAGGTTGA
2B4-R	GAGCCATTCTCCCACTTCAATA
CD27-F	TCAGTGTGATCCTTGCATACC
CD27-R	CGAACGAGAAGACCAGAGTTAC
CD16-1F	GACAATTCCACACAGTGGTTTC
CD16-1R	TTTGTCTGGCACCTGTACTC
Ki67-F	TAACACCATCAGCAGGGAAAG
Ki67-R	CTGCACTGGAGTTCCCATAAA
PRF1-F	CCTGTGAGGAGAAGAAGAAGAAG
PRF1-R	TCGTTAATGGAGGTGTGATGG
GZMA-F	GAGACTCGTGCAATGGAGATT
GZMA-R	CGAGGGTCTCCGCATTTATT
GZMB-F	ACACTCACACACACTACAAGAG
GZMB-R	ACGCACAACTCAATGGTACT
Tbet-F	AAGGATTCCGGGAGAACTTTG
Tbet-R	GTTGGGTAGGAGAGGAGAGTAG
Eomes-1F	GTGGCAAAGCCGACAATAAC
Eomes-1R	CCGAATGAAATCTCCTGTCTCA
STAT4-F	GCTTGGGCATCCATCATTTG
STAT4-R	GTGGCAGGTGGAGGATTATT
NFKB-F	CTCCACAAGGCAGCAAATAGA
NFKB-R	ACTGGTCAGAGACTCGGTAAA
AP1-F	TTCTATGACGATGCCCTCAAC
AP1-R	TCAGGGTCATGCTCTGTTTC
NFAT2-F	AATTCTCTGGTGGTTGAGATCC

NFAT2-R	TACTGGCTTCGCTTTCTCTTC
NFAT1-F	AGTGGCAGAATCGTCTCTTTAC
NFAT1-R	CAGCTGTCTGTGTCTTGTCTT
NFAT5-1F	CGTGGCTCAGTGAAAGATAGAA
NFAT5-1R	AGTCGTTGCCCACAAACA
**ActB-F**	CACTCTTCCAGCCTTCCTTC
**ActB-R**	GTACAGGTCTTTGCGGATGT

Supplementary Table S3: Primer sequences used in Figure 6.

CASP1-F	GCTGAGGTTGACATCACAGGCA
CASP1-R	TGCTGTCAGAGGTCTTGTGCTC
CASP3-F	GGAAGCGAATCAATGGACTCTGG
CASP3-R	GCATCGACATCTGTACCAGACC
CASP8-F	AGAAGAGGGTCATCCTGGGAGA
CASP8-R	TCAGGACTTCCTTCAAGGCTGC
Bcl2-F	ATCGCCCTGTGGATGACTGAGT
Bcl2-R	GCCAGGAGAAATCAAACAGAGGC
MLKL-F	TCACACTTGGCAAGCGCATGGT
MLKL-R	GTAGCCTTGAGTTACCAGGAAGT
RIPK1-F	TATCCCAGTGCCTGAGACCAAC
RIPK1-R	GTAGGCTCCAATCTGAATGCCAG
IL1B-F	CCACAGACCTTCCAGGAGAATG
IL1B-R	GTGCAGTTCAGTGATCGTACAGG
TNR1-F	CCGCTTCAGAAAACCTCAG
TNR1-R	ATGCCGGTACTGGTTCTTCCTG
Atg3-F	ACTGATGCTGGCGGTGAAGATG
Atg3-R	GTGCTCAACTGTTAAAGGCTGCC
LC3B-F	GAGAAGCAGCTTCCTGTTCTGG
LC3B-R	GTGTCCGTTCACCAACAGGAAG
GPX4-F	ACAAGAACGGCTGCGTGGTGAA
GPX4-R	GCCACACACTTGTGGAGCTAGA
**ActB-F**	CACTCTTCCAGCCTTCCTTC
**ActB-R**	GTACAGGTCTTTGCGGATGT
